# A review of qualitative risk assessment in animal health: Suggestions for best practice

**DOI:** 10.3389/fvets.2023.1102131

**Published:** 2023-02-07

**Authors:** Verity Horigan, Robin Simons, Kim Kavanagh, Louise Kelly

**Affiliations:** ^1^Department of Epidemiological Sciences, Animal and Plant Health Agency, Surrey, United Kingdom; ^2^Department of Mathematics and Statistics, University of Strathclyde, Glasgow, United Kingdom

**Keywords:** qualitative, risk, assessment, animal, health

## Abstract

Qualitative risk assessment (QRA) can provide decision support in line with the requirement for an objective, unbiased assessment of disease risk according to the Agreement on the Application of Sanitary and Phytosanitary Measures of the World Trade Organization. However, in order for a QRA to be objective and consistently applied it is necessary to standardize the approach as much as possible. This review considers how QRAs have historically been used for the benefit of animal health, what problems have been encountered during their progression, and considers best practice for their future use. Four main elements were identified as having been the subject of some proposed standard methodology: (i) the description of risk levels, (ii) combining probabilities, (iii) accounting for trade volume and time period, and (iv) uncertainty. These elements were addressed in different ways but were highlighted as being fundamental to improving the robustness in estimating the risk and conveying the results to the risk manager with minimal ambiguity. In line with this, several tools have been developed which attempt to use mathematical reasoning to incorporate uncertainty and improve the objectivity of the qualitative framework. This represents an important advance in animal health QRA. Overall, animal health QRAs have established their usefulness by providing a tool for rapid risk estimation which can be used to identify important chains of events and critical control points along risk pathways and inform risk management programmes as to whether or not the risk exceeds a decision-making threshold above which action should be taken. Ensuring a robust objective methodology is used and that the reasons for differences in results, such as assumptions and uncertainty are clearly described to the customer with minimal ambiguity is essential to maintain confidence in the QRA process. However, further work needs to be done to determine if one objective uniform methodology should be developed and considered best practice. To this end, a set of best practice guidelines presenting the optimal way to conduct a QRA and regulated by bodies such as the World Organization for Animal Health or the European Food Safety Authority would be beneficial.

## Introduction

Risk assessment (RA) is one of the fundamental elements of the risk analysis process alongside hazard identification, risk management and risk communication. It is concerned with the systematic compilation of data/evidence related to an unwanted event, with the objective of providing an evidence base for risk management decisions on how to best mitigate against such an event (1). Since the 1990's, RA has become a useful tool in animal health, for example, import RAs which assess the likelihood of disease introduction *via* international trade of livestock or animal products [e.g., (2, 3)]. The development of such RAs was driven by the need for an objective, unbiased assessment of disease risk in line with the Agreement on the Application of Sanitary and Phytosanitary (SPS) Measures of the World Trade Organization (WTO). Under this agreement all WTO members were required to ensure that their SPS measures were based on an assessment of the risks to human, animal, or plant health taking into account available scientific evidence and using RA methods developed by the World Organization for Animal Health (WOAH, previously OIE). The agreement could thus facilitate trade whilst recognizing that trade cannot be risk free and enable common judgements about the level of risk mitigating measures required. Other uses include microbial risk assessments (MRAs) and/or farm-to-consumption RAs, [e.g., (4, 5)], and rapid veterinary RAs (VRAs), which can form part of a national disease outbreak response. Risk assessments can also perform the role of decision support tools to assist decision makers in the selection and application of the most efficient control and risk mitigation measures during an animal disease outbreak (6, 7).

The WOAH RA framework is based on a model that distinguishes between entry, exposure and consequence assessments (8). For each assessment the main stages typically include: (1) defining the risk question, (2) outlining the steps of the risk pathway, (3) gathering data and information, (4) identifying uncertainty and variability, (5) combining the information in a logical manner, and (6) ensuring the assessment is fully referenced and transparent with reproducible methodology (8). Structuring a RA in this way enables the decision maker to understand the basis of the assessment, its strengths and limitations and allows them to question or provide additional knowledge to improve the assessment (9). Consistency in methods is encouraged by the WOAH, in order to allow for comparison, especially when considering those used for Government decision making (8).

Risk Assessments can be carried out using two general approaches, termed qualitative and quantitative. Qualitative RAs use non-numerical terms to communicate or describe levels of risk, such as high, medium, low, or negligible, whilst quantitative assessments use mathematical calculations to express risks numerically e.g., a risk will occur once every 500 years. Qualitative RAs are often used as an initial screening method to determine the feasibility, needs and data requirements for quantitative RAs. They may also be used in cases where data of sufficient quantity or quality are limited, as they are less demanding in terms of resources and data (10). In situations when rapid decisions are required, such as in an outbreak situation, the speed of conducting a qualitative RA compared to a quantitative counterpart can also be advantageous (11).

This literature review was conducted to evaluate how the use of qualitative RA has progressed in the animal health sphere since the 1990's, by assessing relevant literature, including both reviews and specific case studies. The aim was to identify how methods employed by risk assessors have developed, recognize any standardization of methodologies which have occurred and highlight those areas which still require development to increase the value of qualitative RA.

## Methods

**Figure 1:**
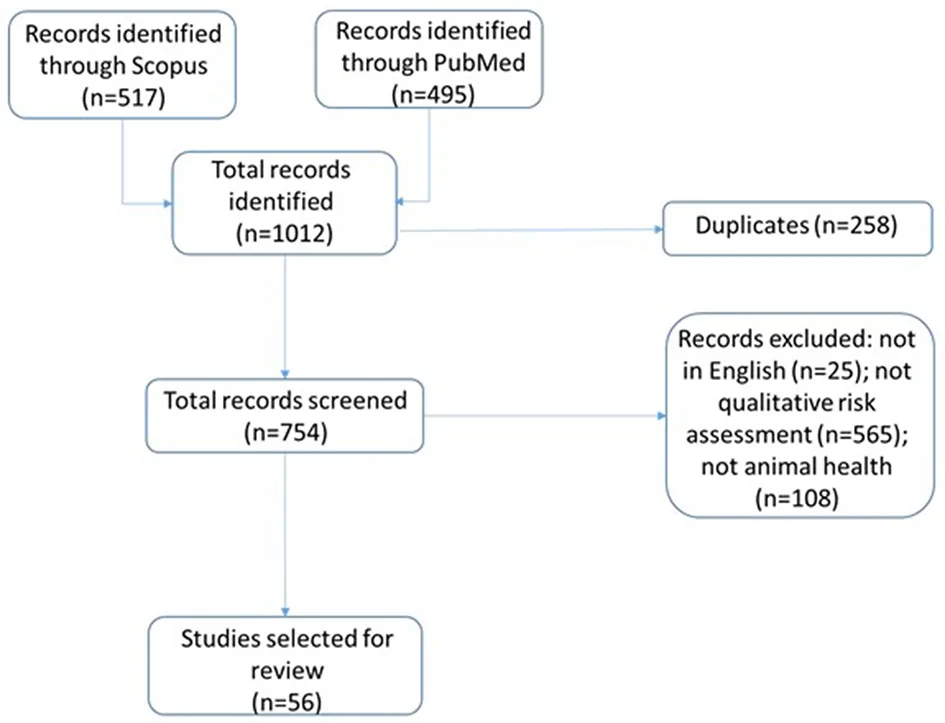
Flow diagram of the decision process and exclusion criteria.

A literature search was conducted in September 2022 in both Scopus (www.scopus.com) and PubMed (www.ncbi.nlm.nih.gov/pubmed) using the search terms “Qualitative and risk and assessment and animal and health” in the “title, keyword, or abstract.” No date range was specified to capture as many articles as possible acknowledging that the field is relatively new. Articles were screened and selected using the exclusion criteria as shown in Figure 1. Initially, any duplicate articles were removed; articles were subsequently included if they were: in English, described qualitative RA and pertained to animal health.

It is acknowledged that no set of search terms will be able to capture all RA articles and that there will inevitably be some that would not have been captured here. Nevertheless, the search terms used were considered optimal as they were found to give the most comprehensive results. It is also acknowledged that the focus on published QRAs is a limiting factor as they are often used by governments for policy decisions and may therefore be less likely to result in publications. Articles concerning risk factors, used to define an “at risk” population, risk management, risk prioritization or risk ranking were excluded. Similarly, animal health was taken as meaning “a pathogen affecting animal health which may result in the importation or transmission of disease *via* either livestock (including fish) or animal/fish products.” Articles referring to food safety risk assessments from a public health perspective were therefore excluded.

## Results

After reviewing the titles and abstracts, 56 articles meeting the inclusion criteria were selected; both reviews (*n* = 15) and case studies (*n* = 41) were included (see Supplementary material for full details). The earliest article selected was from 1993, prior to that year the search results were mostly regarding the application of animal experiment results to human cancer RAs, i.e., they were not concerning animal health *per se*.

**Figure 2:**
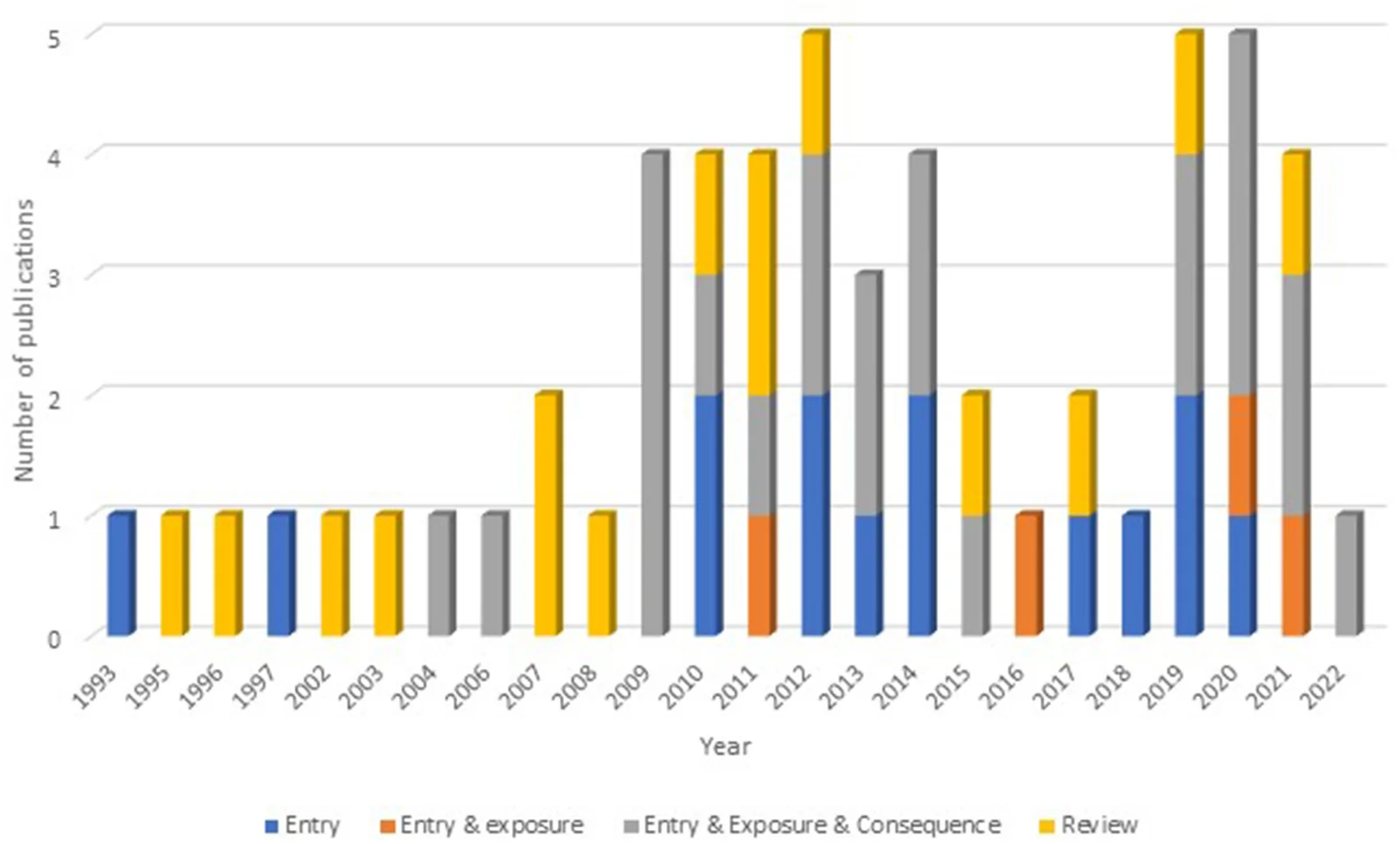
The number and scope of qualitative risk assessments resulting from the search criteria by year.

Out of all the papers reviewed, 25% (*n* = 14) dealt with entry only, i.e., the probability of introduction of a hazard, 7.1% (*n* = 4) covered both entry and exposure and 41.1% (*n* = 23) covered entry, exposure and consequence. A further 26.8% (*n* = 15) were reviews or described a RA tool. The number and scope of the selected articles over time can be seen in Figure 2.

Several of the earliest articles selected were reviews describing the evolution of the qualitative RA process, or elements of it with fundamentals such as defined terminology still under discussion (12, 13). It was noted even in its infancy, however, that for qualitative RA to fulfill its purpose it is necessary to have a common set of methods and technologies. If different methods are used it is possible that disparate risk estimates may be concluded from the same evidence (12).

There were six themes identified from this review as being fundamental to the operation of a robust qualitative RA that are described in detail in the following sections. The first theme was how to communicate the meaning of the terms describing the probability of occurrence at each step of the risk pathway in a standard manner. Secondly, was how to combine these probability terms to give an overall estimate of risk. Another topic highlighted was how to address the uncertainty associated with the probability estimates of the risk pathway steps. In particular, how to derive an overall uncertainty level, and how to make the influence uncertainty can have on the overall risk estimate clear to the decision maker. The topics of dose response and accounting for multiple products/animals (aggregated probability) were discussed by only a limited number of articles (*n* = 11) but are important aspects of risk and are highlighted here as topics for future consideration. Finally, four articles discussed the use of semi-quantitative tools which have been developed to convert the descriptive terms in qualitative RA pathways into numerical values.

### Description of terms—definition of risk levels

In a qualitative RA the level of risk is communicated to the decision maker, or risk manager, by assigning a certain number of descriptive risk levels with associated definitions. The terms need to be well-defined, and it is important to interpret individual qualitative RA results in light of whatever specific categorical definitions are used (14, 15). For example, an overall risk estimate of “low” can be meaningless to a risk manager without some sort of indication of what this definition constitutes in the eyes of the author of the risk assessment (16).

The definitions of the different risk levels varied in the articles reviewed here (Table 1); the most commonly used were those defined by the European Food Safety Authority (EFSA) (21). The number of levels used ranged from 4 to 7 with all articles using the levels “negligible,” “low,” “medium,” and “high” and only some using “extremely low,” “very low,” and “very high.” Whilst the EFSA (2006) definitions were predominantly used, it was noted that some of the examples relate to the frequency of repeated events or outcomes (e.g., often, regularly) and some to the likelihood of a single event or outcome (16). Additionally, although the term “negligible” is commonly used in the risk assessment terminology, it can be perceived as having a risk management connotation in everyday language. Clarification may therefore be needed to avoid the impression that risk assessors are making risk management judgments (16).

**Table 1:** Summary of the definitions of qualitative risk/probability levels used/defined in the papers selected through the literature review.

**Original definitions first published**	**(17)**	**(18)**	**(19)**	**(20)**	**(9)**	**(21)**	**(22)**	**(23)**	**(24)**
Subsequently used in these risk assessments	(14, 25–27)	(28, 29)	(15, 30)	(31)	(9)	(32–41)	(6)	(42)	(43)
Very high	Almost certain to occur					Event occurs almost at certainly			
High	Expected to occur	Occurrence of the event is clearly a possibility (probable)	When exposure or transmission is likely to occur	Extending above the normal of average level	Occurrence of event is clearly a possibility	Occurs (very) often	An event is almost certain to occur	Likelihood of an event occurring is very often	The event would be very likely to occur
Moderate	Less than 50:50 probability	Occurrence of the event is a possibility (in the majority of cases)	When exposure or transmission may occur in all cases	The usual amount, extent, rate	Occurrence of event is a possibility	Occurs regularly	An event is likely to occur with a high probability	Likelihood of an event occurring is regular	The event would occur with an even probability
Low	Unlikely to occur	Occurrence of an event is a possibility in some (a minority of) cases	When exposure or transmission may occur in some cases	Less than average, coming below the normal level	Occurrence of event is a possibility in some cases	Rare but could occur	An event is unlikely to occur	Likelihood of an event occurring is occasional	The event would be unlikely to occur
Very low	Rarely occur					(Very) Rare but cannot be excluded	An event is very unlikely to occur	Likelihood of an event occurring is rare but does occur	The event would be very unlikely to occur
Extremely low	Very rarely occur							Likelihood of an event occurring is extremely rare but cannot be excluded	The event would be extremely unlikely to occur
Negligible	Chance of occurrence so small it can be ignored	Probability of occurrence of the event is possible only in exceptional circumstances (or sufficiently low to be ignored)	When the probability of exposure or transmission is sufficiently low to be ignored, or if the event is possible only in exceptional circumstances	Not worth considering; insignificant	Probability of event sufficiently low to be ignored or event only possible in exceptional circumstances	So rare that it does not merit consideration	An event virtually would not occur	Likelihood of an event occurring is so rare that it does not merit consideration	The event would almost certainly not occur

Several articles drew attention to the clustering of risk at the lower end of the descriptive scale and the potential benefit of the inclusion of additional levels to extend the range of adjectives used for the lower probabilities in order to provide greater detail (10, 15). One article suggested the use of a 10-point ordinal scale with corresponding adjectives such as “null,” “nearly null,” “minute,” “extremely low,” etc. However, because of the difficulty of gaining universal agreement on specific definitions of the words, it proved challenging to define each word precisely (10). Another complication highlighted was that differentiating between “low,” “very low,” and “extremely low” in these circumstances may be considered arbitrary and, as such, add a further level of uncertainty to the RA (15).

Analysis of 219 opinions published by the Scientific Panels revealed that RA terminology is not fully harmonized within EFSA. This was caused, in part, by sectoral legislation defining specific terminology and international standards for specific fields of RA and thus for specific panels (16). In order to reduce ambiguity, the Scientific Committee recommended that Scientific Panels should, wherever possible, work toward quantitative expression of the probability of the adverse effect and of any quantitative descriptors of that effect (e.g., duration). For example, in a United Kingdom (UK) Non-native Organism RA, likelihood levels for entry and establishment were defined for events over a 5 year period using the following quantitative terms (Table 2).

**Table 2:** UK Non-native Organism Risk Assessment scheme likelihood descriptors for entry and establishment (44).

Likelihood levels	Chance of occurrence over a 5 year period
Very unlikely	<10% chance of occurring
Unlikely	10–33% chance of occurring
Moderately likely	33–66% chance of occurring
Likely	66–90% chance of occurring
Very likely	>90% chance of occurring

*Suggested best practice: Harmonized and consistent use of one set of definitions e.g., EFSA* (21). *The clustering of risk at the lower end of the scale requires further development*.

### Combining probabilities

The majority of the risk pathways described in the articles reviewed, were designed as a series of multiplicative conditional probabilities i.e., each step in the pathway has to occur in order for the next step to be possible. As such, the likelihood level for each step of the pathway is independent of the previous step and combining these levels cannot lead to an increase in risk. However, there was not always transparency on how the probabilities of the events were combined.

There is not a universally recognized standard methodology to combine the probabilities of each step of a risk pathway, or across the risk assessments steps (entry, exposure, and consequence), to produce and communicate an overall estimate. As such, it was not surprising to find that the articles reviewed used different methods. One way of visually demonstrating how to combine risk levels is by using risk matrices, which have previously been used in RAs to combine the probability and impact of an event occurring to give the overall risk level [see (45) for an overview]. Matrices provide a transparent methodology for combining risk levels and help decision-makers to focus on the highest priority risks. However, the approach doesn't always account for all considerations in more complex assessments, such as the volume of a product being imported or issues around combining a large number of probabilities using the same matrix. As such, it has been suggested that they should only be used to illustrate results rather than as calculators of likelihood or risk (43).

Published matrices using a multiplicative risk framework varied in the RAs reviewed here. One of the most commonly used adheres to the principle that the product of two probabilities is always equal to the lowest probability (Table 3) (6, 9, 30, 40, 42, 46). This matrix defines a likelihood estimate for any binary combination of conditional events but does not allow for the product of multiple conditional probabilities to be lower than the lowest value of the individual probabilities.

**Table 3:** When combining two probabilities, the resulting probability is not greater than the lower probability scale of the two (42) [from Dufour et al. (10)].

**Probability**	**Negligible**	**Extremely low**	**Very low**	**Low**	**Medium**	**High**
Negligible	Negligible	Negligible	Negligible	Negligible	Negligible	Negligible
Extremely low	Negligible	Extremely low	Extremely low	Extremely low	Extremely low	Extremely low
Very low	Negligible	Extremely low	Very low	Very low	Very low	Very low
Low	Negligible	Extremely low	Very low	Low	Low	Low
Medium	Negligible	Extremely low	Very low	Low	Medium	Medium
High	Negligible	Extremely low	Very low	Low	Medium	High

This matrix has been further developed to allow for an improved estimation of risk when multiplying more than two conditional probabilities and takes into account that the product of probabilities that are assessed to be “low” or “very low” will likely be lower than the lowest individual probability (Table 4) (38) but could underestimate the risk for a small number of probabilities. Further expansion of this idea shows the product of two probabilities to be usually less than the lowest probability (and sometimes given as a range; Table 5).

**Table 4:** Expanded risk matrix to account for the product of two “low” probabilities being less than the lowest probability (38).

**Probability**	**Negligible**	**Very low**	**Low**	**Medium**	**High**	**Very high**
Negligible	Negligible	Negligible	Negligible	Negligible	Negligible	Negligible
Very low	Negligible	Negligible	Negligible	Very low	Very low	Very low
Low	Negligible	Negligible	Very low	Low	Low	Low
Medium	Negligible	Very low	Low	Medium	Medium	Medium
High	Negligible	Very low	Low	Medium	High	High
Very high	Negligible	Very low	Low	Medium	High	Very high

**Table 5:** Expanded risk matrix to account for the product of two probabilities being less than the lowest probability (27).

**Probability step *“n+1”***	**Conditional probability step “** ***n*** **”**					
	**Negligible**	**Extremely low**	**Very low**	**Low**	**Moderate**	**High**
Negligible	Negligible	Negligible	Negligible	Negligible	Negligible	Negligible
Extremely low	Negligible	Negligible	Negligible-extremely low	Extremely low	Extremely low	Extremely low
Very low	Negligible	Negligible-very low	Extremely low	Extremely low	Very low	Very low
Low	Negligible	Extremely low	Extremely low	Very low	Very low	Low
Moderate	Negligible	Extremely low	Very low	Very low	Low	Moderate
High	Negligible	Extremely low	Very low	Low	Moderate	Moderate

In the event that two or more independent factors contribute to the probability estimation for a single pathway step, the probability for each factor can be estimated by considering them as being additive rather than conditional probabilities that should be multiplied. Risk matrices for such combinations can also be developed to show the overall probability for that step (27). Alternatively, the factor with the “worst” estimate for a specific step can be selected (9). This method, however, does not acknowledge that in some cases the assessment of one factor may dominate and determine the overall assessment in which case it may be necessary to combine the overall risk in a more complicated fashion, for example, using weightings, etc. (47).

Wieland et al. (9) takes this one step further and provides a matrix for combining probabilities of independent steps where an increase of the overall risk is possible between steps, for example spread of disease leads to an increased number of infected animals and therefore to an increased overall risk. The matrix averaged the risk estimates of independent steps and was based on one developed by Zepeda (18) (Table 6).

**Table 6:** Combination matrix, used to combine two risk estimates that are independent of each other and/or where an increase of risk is possible.

	**Results of the assessment of parameter 2**			
**Results of the assessment of parameter 1**	**Negligible**	**Low**	**Moderate**	**High**
Negligible	Negligible	Low	Low	Moderate
Low	Low	Low	Moderate	Moderate
Moderate	Low	Moderate	Moderate	High
High	Moderate	Moderate	High	High

This matrix's principle transfers the average of independent probabilities to combinations of qualitative risk levels [based on Zepeda (18)].

Overall, the choice of an appropriate risk matrix varied between qualitative RAs, with some authors arguing that a limited number of risk categories can result in a general over-estimation of the risk and a low resolution overall. Whilst an increased number of risk categories can increase the resolution of the RA, introducing more risk levels can reduce the accuracy/confidence about the final estimate if there is limited data or high uncertainty. As Tables 4–6 demonstrate, it is not inherently incorrect for a risk assessor to develop a bespoke risk matrix for a given RA, it is much more important that the matrix is applied consistently, transparently and is appropriate for the risk pathway.

Some authors deliberately chose not to use a risk matrix approach for combining probabilities concluding that they can give a false impression of scientific robustness, whilst actually relying on subjective risk level estimates which may be influenced by a range of other considerations such as personal knowledge and beliefs (15, 32). They also highlighted the issue of the inability to account for marked variation in estimates within categories, and loss of information with successive levels of multiplication. As a result, they preferred to use a qualitative descriptive approach that allowed them to conclude an overall risk level and highlight areas of particular uncertainty and variability.

*Suggested best practice: Transparent use of a specified matrix for conditional probabilities. Further development is required to take into account that the product of probabilities that are assessed to be “low” or “very low” will likely be lower than the lowest individual probability. For independent factors, an additive approach should be taken with the use of weightings to identify the contribution of these factors to the overall risk level*.

### Consideration of trade volume and time period

For the qualitative RAs reviewed here, risk levels were often assessed on a per product/animal basis, with no consideration being made of the impact if multiple products/animals were assessed. Such an impact can be significant if the number of products/animals is very high. WOAH guidance (8) indicates that the volume should be taken into account but offers no detailed methodology on how to do this. A RA that does not specify the volume of products/animals, constrains its use to determine mitigation measures to reduce risk to an acceptable level (43). Thus, the transparency and defensibility of a qualitative RA are enhanced by defining both the unit and volume of products/animals.

Consideration of the volume of trade was mentioned descriptively in some articles. For example, one article on the risk of introducing peste des petits ruminants into Tanzania used a questionnaire to estimate the amount of trade along the border area between Zambia and Tanzania. Responses led them to conclude that “the probability of entry as determined by trade volume was rated low” (29). Peel et al. (44) found that the imprecise monitoring of live amphibians into the UK meant that on further investigation the undeclared volume of amphibian trade into the UK, was sufficiently large to make the introduction of *batrachochytrium dendrobatidis* into the UK natural environment very likely under current systems.

Similarly, the probability of Henipavirus entering the UK was assessed by combining the number of products imported annually (*N*) with the results from the probability pathway (*P*) which assesses the probability per animal, human or ton of foodstuff using a non-matrix approach, i.e., assessing each combination of *N* and *P* on a case-by-case basis. It was assumed that if the number of imports (*N*) was negligible, then the probability of entry was also negligible (34).

A qualitative RA for entry of highly pathogenic avian influenza (HPAI) strain H5N1 into the UK accounted for increased risk due to the number of birds migrating from different regions of the world with different pathogen prevalence (38). Although the predicted probabilities of entry of H5N1 per individual bird per year were low, very low or negligible, the overall risk was high for a few species reflecting the high numbers of birds migrating from some regions. The number of birds was addressed qualitatively but with comparable numerical values i.e., >1,000,000 very high; 100,001–1,000,000 high; 10,001–100,000 medium; 1,001–10,000 low; 1–1,000 very low; 0 negligible (38).

A key paper addressing the aggregated probability for qualitative RA communicating the risk per year or per unit of product concluded that it was essential to phrase the risk question to account for aggregated risk, whether due to volume of trade or length of time period (48). The assumption in many RAs is that units are independent and have the same probability of being infected. For the higher levels of probability (very high, high, and medium) this is logical because if an individual unit is likely to be infected/contaminated then a group of units will also have a high chance of being infected/contaminated. However, for the lower levels of probability, if the volume is high enough, the aggregated risk could be under-estimated, that is, assessed as being of a lower qualitative category of risk than is probably realistic.

Given an individual risk level and volume of product the estimated values of aggregated probability can be derived from a contour plot [see (48)] and then be used to give guidance on the likely level of qualitative risk. Even though this application relies on making assumptions concerning the individual probability it can give an idea of the possible magnitude of the aggregated probability and provide a range of uncertainty around it. The contour plot relies on quantitative bounds used for the qualitative levels and results are therefore dependent on the choice of these bounds with different results likely being derived for different values.

The aggregated probability method described by Kelly et al. (48) has been applied to two RAs (34, 49) with the assumption that the aggregated probability calculations used the same quantitative bounds as used in the tool by Kelly et al. (48) acknowledging that this probability could change if these bounds were to be altered. For the RA previously described on the risk of introduction of henipavirus into the UK (34), it was found that for the lower categories of individual probability, the number of imports was important in determining whether or not the aggregated probability is of a higher qualitative level than the individual probability. Overall, the results were consistent between the two methods, identifying the imported commodity with the highest associated risk. However, whilst the methodology adopted by Snary et al. (34) provided results that clearly highlight the routes of highest risk, the evaluation of the aggregated risk was not as transparent as the method described by Kelly et al. (48).

*Suggested best practice: The risk question should be phrased to account for the number of units or time period, not on a per product basis. The use of a specified metric such as the contour plot developed by Kelly et al*. (48)*, should be used. This metric should also be further explored with regard to the effect of using different quantitative bounds*.

### Uncertainty

The concept of risk involves uncertainty in both the likelihood of occurrence and the magnitude of the consequences. Uncertainty in risk estimates can stem from lack of data, biological variation (reflecting true ranges and variability in biological systems) and measurement error (50). Reducing the amount of uncertainty does not necessarily change the actual risk but gives a more precise evaluation of it, thereby giving more confidence in the risk assessment outputs (12). This is particularly important where, within the range of uncertainty, the risk estimate could potentially surpass a key decision-making threshold (51).

For qualitative RA the dilemma is how to deal with uncertainty so that it is clear to the decision maker where it exists and how it may influence the overall risk estimate. If done well, characterization of uncertainty is a beneficial aspect of qualitative RA, as it emphasizes the importance of uncertainty and can include guidance on its management (50). Such assessments are also beneficial in identifying data gaps as a result of recognizing where areas of uncertainty exist. Some of the review papers assessed here stated that more comprehensive guidance is needed, firstly on the assessment and reporting of uncertainty and secondly on the use of uncertainty estimates when judging assessments against acceptable levels of risk (43).

Providing the uncertainty level of all estimates can make a RA more transparent and accessible for risk managers. Several articles provided descriptive levels of uncertainty in a similar manner to that of the likelihood definitions as shown in Table 2. Risk managers are then able to identify which steps drive the risk in the model and what results need to be interpreted with care due to high uncertainty (9). However, few articles mentioned, or dealt with, how to estimate an overall level of uncertainty associated with the overall risk estimate. In one example, the highest uncertainty estimate was selected along the steps of the pathways so a high uncertainty in any one level led to a high uncertainty in the overall outcome. An exception was made if the occurrence of an event was Negligible with Low uncertainty (27).

The use of expert opinion was described by some authors to reduce the uncertainty surrounding parameters where data were scarce (7, 30, 39, 41, 42, 52). Some studies used workshops involving experts from a range of relevant backgrounds to confirm risk parameters, risk pathways and numerical weightings for risk factors reaching a final consensus of agreement (7, 52). Additional studies employed the Delphi technique (39, 42) to reach consensus. One study used the level of disagreement between different experts as an indicator of the level of uncertainty (39). Qualitative risk estimates were transformed into quantitative scores (negligible = 1; very high = 6) and then the average of the absolute difference of individual risk estimates to the mode was calculated. The resulting averages were ranked and subjective cut-offs for three uncertainty levels were defined. The purpose of these categories was mainly for communication reasons.

Finally, one article used numerical terms to describe uncertainty as confidence levels by using associated levels of the chance of being correct (Table 7).

**Table 7:** UK Non-native Organism Risk Assessment (NNRA) scheme: confidence descriptors for uncertainty levels (44).

**Confidence descriptor**	**Associated level of chance**
Low	~35% chance or less of being correct
Medium	~50% chance of being correct
High	~80% chance of being correct
Very high	~90% chance or better of being correct

*Suggested best practice: The definitions of uncertainty levels and method of calculation should be clearly defined. Risk assessors should be transparent in their decision to either identify at which stages the highest uncertainty exists or whether to give an overall uncertainty level. Further development of how to calculate an overall uncertainty level is required e.g., whether the assessor uses the highest uncertainty level along the risk pathway or the uncertainty level associated with the pathway step that decides the overall risk level*.

### Dose-response

Risk is often viewed as a binary outcome of entry and exposure in qualitative RAs and does not take into account the amount of pathogen released which may not always be sufficient for infection to occur (43). As such, any qualitative RA that considers infection should assess not only the likelihood of exposure to a pathogen, but also the level of pathogen exposure (31, 43). The behavior of any pathogen throughout the risk pathway will vary according to the type of pathogen being assessed and whether it is in the live animal or on an animal product. Whether or not infection occurs will subsequently depend on the animal which is exposed to the pathogen and whether it has prior immunity for example (14).

Articles that used qualitative methods for addressing the level of pathogen were limited. Those that did had varying approaches, for example, purely descriptive (31, 46), qualitatively evaluating the risk pathways whilst using quantitative evaluations of the level of pathogens (53) and considering the reduction in viral load using a matrix approach (54). The latter paper estimated the probability of avian influenza virus survival on different types of equipment before and after preliminary and secondary cleansing and disinfection (C&D) procedures after an outbreak. A risk matrix spreadsheet tool identified those areas of the house which may still contain sufficient virus post-preliminary C&D for infection to occur and on which attention should be focussed during secondary C&D (54).

*Suggested best practice: A RA should assess both the likelihood of exposure to a pathogen and the level of pathogen exposure. This area needs to be fully explored before specific best practice methodology can be advised*.

### Tools of the trade

Some articles described tools, or models, which have been developed to convert descriptive levels used in qualitative RAs into numerical values and so able to use mathematical probabilities to calculate the risk in quantitative terms (55–58). The process is then reversed to conclude with an overall risk estimate in qualitative descriptive terms. These tools are often termed semi-quantitative with respect to their use of numerical values.

de Vos et al. (58) compared generic risk models that can be applied to assess the incursion risk for multiple animal diseases. Of the seven tools assessed, four were semi-quantitative [RRAT (59), MINTRISK (60), IDM (61), and NORA (57)] and one was qualitative [SVARRA (62)]. All the tools were primarily based on the WOAH import RA framework (8). The tools varied in their approaches to uncertainty, MINTRISK and SVARRA explicitly asked the risk assessor for their assessment of uncertainty in estimating the input parameter values, but MINTRISK additionally used stochastic simulation to assess uncertainty.

The main algorithms used in MINTRISK were sampling from triangular distributions on a linear scale between 0 and 1, these were then translated into qualitative risk scores for each step in the model and for the overall risk estimate. The method developed by Australian Biosecurity (56) is similar to that of MINTRISK but a uniform distribution was used. With NORA, the combination of values within a pathway were calculated by applying the basic probability calculation rules of serial (multiplying) and parallel (summing) processes. As an output the final numerical value of probability was then converted into a “verbal score” (descriptive risk level). This conversion was for the purpose of inclusivity acknowledging that “some people merely like to see numbers, while others need to have a verbal score to feel comfortable with the answers.” As mentioned in the description of terms section, the same verbal score can actually mean a different risk level for different people and so definitions of the verbal scores for probability and impact are included in the NORA guidebook. The tool developers also caveat that the location of the qualitative definitions within the numerical class should be taken into account as the estimated risk might be close to a limit between two classes and is therefore relatively sensitive to small changes in input values (57).

These semi-quantitative tools are still qualitative RAs but have introduced the concept of mathematical principles to standardize their approach. No automated tools were captured in this literature review which addressed the aggregated probability in an entry assessment or the level of pathogen in an exposure assessment.

*Suggested best practice: These should be explored further, specifically the use of probability distributions to take uncertainty into account and the incorporation of the volume of trade to give an overall risk level*.

## Discussion

This review set out to investigate the progression of qualitative RA in animal health and to identify the main themes that have been explored and addressed as the method has evolved. The qualitative RA methodology was chosen in several case studies reviewed here because it was perceived as being simple to conduct, easy to communicate and helped to provide credibility to the work due to being an accepted methodology for customers and policy makers. Furthermore, a qualitative RA is ideal for identifying important chains of events and critical control points along risk pathways which can then be used to construct robust and informed risk management programs.

It should be acknowledged that this review did not cover gray literature and only those articles which had undergone a peer review process and were available *via* the search engines PubMed and Scopus were included. This may underreport the general “usefulness” of qualitative RAs which are very often used by governments for rapid policy decisions and may be less likely to result in published articles. As a general estimate the authors consider that between 70 and 80% of qualitative RAs commissioned by governments may go unpublished. However, as these RAs are not published on gray literature search sites and may not be publicly available on government websites it is not possible to verify this estimate.

The four main elements of qualitative RA that were identified in this review as having been the subject of some proposed standard methodology were (i) the description of risk levels, (ii) combining probabilities, (iii) treatment of aggregated probabilities, and (iv) uncertainty. These elements were addressed in different ways by the articles reviewed but were highlighted as being fundamental to improving the accuracy in estimating the risk and conveying the results of the RA to the risk manager with minimal ambiguity. The development of standardization of methodology thus represents an important advance in qualitative animal health RA.

Despite these developments a few key challenges remain. Further work needs to be done regarding an objective uniform methodology for deriving an overall uncertainty and risk estimate. More thought also needs to be given to improve the perceived robustness of qualitative RAs. Ensuring a robust objective methodology is used and that the reasons for differences in results, such as assumptions and uncertainty are clearly described to the customer is essential to maintain confidence in the qualitative RA process. One way of doing this is by adopting some of the characteristics of a quantitative analysis (10), as has been shown by the development of semi-quantitative tools such as NORA and MINTRISK.

Guidelines for RA in international trade have been published by the WOAH (8), but little detail is provided about how to use qualitative methods in practice and several solutions were proposed to address this across the papers reviewed. This is in line with the need for RA to remain flexible to deal with real life scenarios, recognizing that no single approach may be applicable in all cases. Despite this, it can be concluded that some level of standardization is important to help prevent discrepancies in results due to the broad approaches used. A set of best practice guidelines set out by a body such as WOAH or EFSA would be beneficial to establishing a standard methodology for conducting qualitative RAs. Preliminary suggestions for current best practice based on the findings from this review and areas where best practice is yet to be substantiated have been identified for all of the themes discussed here.

In summary, the robustness of conclusions from qualitative RAs has improved since the 1990's with the introduction of consistent definitions of probability terms, risk pathways, tabulated matrices illustrating the combination of conditional probabilities, methods to assess the aggregated probability and consideration as to how uncertainty can be addressed. Several tools have been created which apply mathematical reasoning by allowing for uncertainty to be accounted for and for the probabilities of the risk pathway steps to be combined. Overall animal health qualitative RAs have established their usefulness by providing a tool for rapid risk estimation which can be used to identify whether or not risk exceeds a decision-making threshold above which action should be taken. Based on this review, future directions should include further development of a uniform methodology for deriving an overall uncertainty estimate and further improvement to the standardized methodologies employed to maintain confidence in the qualitative RA process.

## Author contributions

VH, KK, RS, and LK contributed to conception of the review. VH wrote the first draft of the manuscript. All authors contributed to manuscript revision, read, and approved the submitted version.
