# Supplementary material for: A review of qualitative risk assessment in animal health: Suggestions for best practice
Source: Front Vet Sci. 2023 Feb 7;10:1102131. doi: 10.3389/fvets.2023.1102131 (PMC9941190; doi:10.3389/fvets.2023.1102131)
Supplement: Supplementary file 1 [file Table_1.DOCX]

# Supplementary material

**Table S1. Articles meeting the selection criteria for further analysis by manuscript type and classification by study.**

| **Title** | **Year** | **Journal** | **Manuscript type** | **Classification by study** |
| --- | --- | --- | --- | --- |
| An assessment of the risk of foreign animal disease introduction into the United States of America through garbage from Alaskan cruise ships. | 1993 | Revue scientifique et technique (International Office of Epizootics) | Case study | Entry |
| Risk analysis systems for veterinary biologicals: a regulator's tool box. | 1995 | Revue scientifique et technique (International Office of Epizootics) | Review | N/A |
| The application of probabilistic scenario analysis for risk assessment of animal health in international trade | 1996 | Annals of the New York Academy of Sciences | Review | N/A |
| The potential risks to animal health from imported sheep and goat meat. | 1997 | Revue scientifique et technique (International Office of Epizootics) | Case study | Entry |
| Health risk assessment of the translocation of wild animals | 2002 | OIE Revue Scientifique et Technique | Review | N/A |
| Risk assessments on BSE [3] | 2003 | Veterinary Record | Review | N/A |
| Qualitative analysis of the risk of introducing Gyrodactylus salaris into the United Kingdom | 2004 | Dis Aquat Organ | Case study | Entry, Exposure & Consequence |
| Evaluation of bovine spongiform encephalopathy (BSE) infection risk of cattle via sewage sludge from wastewater treatment facilities in slaughterhouses in Japan | 2006 | Journal of Veterinary Medical Science | Case study | Entry, Exposure & Consequence |
| Application of risk assessment and decision analysis to aquatic nuisance species. | 2007 | Integrated environmental assessment and management | Review | N/A |
| The application of risk analysis in aquatic animal health management | 2007 | Preventive Veterinary Medicine | Review | N/A |
| Mapping the future dynamics of disease transmission: risk analysis in the United Kingdom Foresight Programme on the detection and identification of infectious diseases. | 2008 | Euro surveillance : bulletin européen sur les maladies transmissibles = European communicable disease bulletin | Review | N/A |
| Tool for Estimating the Risk of Anthropogenic Spread of Batrachochytrium denrobatidis Between Water Bodies. | 2009 | EcoHealth | Case study | Entry, Exposure & Consequence |
| Assessment of exotic fish disease introduction and establishment in the United Kingdom via live fish transporters | 2009 | Diseases of Aquatic Organisms | Case study | Entry, Exposure & Consequence |
| Epizootic Haematopoietic Necrosis Virus - An Assessment of the Likelihood of Introduction and Establishment in England and Wales. | 2009 | Preventive Veterinary Medicine | Case study | Entry, Exposure & Consequence |
| A qualitative risk assessment of factors contributing to foot and mouth disease outbreaks in cattle along the western boundary of the Kruger National Park | 2009 | OIE Revue Scientifique et Technique | Case study | Entry, Exposure & Consequence |
| Risk of equine infectious disease transmission by non-race horse movements in Japan | 2010 | Journal of Veterinary Medical Science | Case study | Entry |
| Qualitative assessment of the commodity risk for spread of foot-and-mouth disease associated with international trade in deboned beef | 2010 | Transboundary and Emerging Diseases | Case study | Entry |
| Qualitative risk assessment of the acquisition of Meticillin-resistant staphylococcus aureus in pet dogs | 2010 | Risk Anal | Case study | Entry, Exposure & Consequence |
| The feasibility of developing a risk assessment for the impact of climate change on the emergence of Crimean-Congo haemorrhagic fever in livestock in Europe: A Review | 2010 | Journal of Applied Microbiology | Review | N/A |
| Qualitative risk assessment in a data-scarce environment: A model to assess the impact of control measures on spread of African Swine Fever | 2011 | Preventive Veterinary Medicine | Case study | Entry, Exposure & Consequence |
| Risk assessment of the introduction of Rift Valley fever from the Horn of Africa to Yemen via legal trade of small ruminants | 2011 | Trop Anim Health Prod | Case study | Entry and exposure |
| A qualitative risk assessment methodology for scientific expert panels | 2011 | OIE Revue Scientifique et Technique | Review | N/A |
| Comparison of veterinary import risk analysis studies | 2011 | International Journal of Risk Assessment and Management | Review | N/A |
| Geographical BSE risk assessment and its impact on disease detection and dissemination | 2012 | Preventive Veterinary Medicine | Case study | Entry |
| Risk Assessment of the Introduction of H5N1 Highly Pathogenic Avian Influenza as a Tool to be Applied in Prevention Strategy Plan | 2012 | Transboundary and Emerging Diseases | Case study | Entry, Exposure & consequence |
| Qualitative risk analysis of introducing batrachochytrium dendrobatidis to the UK through the importation of live amphibians | 2012 | Diseases of Aquatic Organisms | Case study | Entry, Exposure & consequence |
| Qualitative release assessment to estimate the likelihood of henipavirus entering the United Kingdom | 2012 | PLoS One | Case study | Entry |
| Scientific Opinion on Risk Assessment Terminology | 2012 | EFSA Journal | Review | N/A |
| Qualitative risk assessment for the endemisation of Dirofilaria repens in the state of Brandenburg (Germany) based on temperature-dependent vector competence | 2013 | Parasitology Research | Case study | Entry, Exposure & consequence |
| Risk assessments of lumpy skin diseases in Borena bull market chain and its implication for livelihoods and international trade | 2013 | Tropical Animal Health and Production | Case study | Entry, Exposure & Consequence |
| A risk and benefit assessment for visual-only meat inspection of indoor and outdoor pigs in the United Kingdom | 2013 | Food Control | Case study | Entry |
| A qualitative risk assessment for visual-only post-mortem meat inspection of cattle, sheep, goats and farmed/wild deer | 2014 | Food Control | Case study | Entry |
| Entry of H5N1 highly pathogenic avian influenza virus into Europe through migratory wild birds: A qualitative release assessment at the species level | 2014 | Journal of Applied Microbiology | Case study | Entry |
| A qualitative assessment of the risk of introducing peste des petits ruminants into northern zambia from Tanzania | 2014 | Vet Med Int | Case study | Entry, Exposure & Consequence |
| Do imports of rainbow trout carcasses risk introducing viral haemorrhagic septicaemia virus into England and Wales? | 2014 | Transboundary and Emerging Diseases | Case study | Entry, Exposure & Consequence |
| Foot and mouth disease risk assessment in Mongolia-Local expertise to support national policy | 2015 | Preventive Veterinary Medicine | Case study | Entry, Exposure & Consequence |
| Animal Disease Import Risk Analysis - a Review of Current Methods and Practice | 2015 | Transboundary and Emerging Diseases | Review | N/A |
| Qualitative risk assessment of introduction of anisakid larvae in Atlantic salmon (Salmo salar) farms and commercialization of products infected with viable nematodes | 2016 | Food Control | Case study | Entry and Exposure |
| Assessment of the risk of African swine fever introduction into Finland using NORA—a rapid tool for semiquantitative assessment of the risk | 2017 | Transboundary and Emerging Diseases | Case study | Entry |
| A Comparison of Disease Risk Analysis Tools for Conservation Translocations | 2017 | EcoHealth | Review | N/A |
| Qualitative import risk assessment: A proposed method for estimating the aggregated probability of entry of infection | 2018 | Microbial Risk Analysis | Case study | Entry |
| The Risk of Foot and Mouth Disease Transmission Posed by Public Access to the Countryside During an Outbreak | 2019 | Frontiers in Veterinary Science | Case study | Entry, Exposure & Consequence |
| Risk assessment on rabies entry through hunting dog movement with semi-quantitative approach to Sumatera Island, Indonesia | 2019 | Journal of Advanced Veterinary and Animal Research | Case study | Entry |
| Introduced deer and their potential role in disease transmission to livestock in Australia | 2019 | Mammal Review | Case study | Entry |
| A qualitative risk assessment of cleansing and disinfection requirements after an avian influenza outbreak in commercial poultry | 2019 | Br Poult Sci | Case study | Entry, Exposure & Consequence |
| Risk assessment of exotic disease incursion and spread | 2019 | EFSA Journal | Review | N/A |
| A Qualitative Risk Assessment of Rabies Reintroduction Into the Rabies Low-Risk Zone of Bhutan | 2020 | Frontiers in Veterinary Science | Case study | Entry, Exposure & Consequence |
| Using a participatory qualitative risk assessment to estimate the risk of introduction and spread of transboundary animal diseases in scarce-data environments: A Spatial Qualitative Risk Analysis applied to foot-and-mouth disease in Tunisia 2014-2019 | 2020 | Transboundary and Emerging Diseases | Case study | Entry and exposure |
| Cross-Validation of Generic Risk Assessment Tools for Animal Disease Incursion Based on a Case Study for African Swine Fever | 2020 | Front Vet Sci | Case study | Entry, Exposure & Consequence |
| Qualitative risk assessment of transmission pathways of highly pathogenic avian influenza (HPAI) virus at live poultry markets in Dhaka city, Bangladesh | 2020 | Zoonoses Public Health | Case study | Entry, Exposure & Consequence |
| Assessing the aggregated probability of entry of a novel prion disease agent into the United Kingdom | 2020 | Microb Risk Anal | Case study | Entry |
| The risk from SARS-CoV-2 to bat species in england and mitigation options for conservation field workers | 2021 | Transboundary and Emerging Diseases | Case study | Entry, Exposure & Consequence |
| A qualitative risk assessment indicates moderate risk of foot-and-mouth disease outbreak in cattle in the lower Okavango Delta because of interaction with buffaloes | 2021 | Transbound Emerg Dis | Case study | Entry, Exposure & Consequence |
| Stay alert: probability of African Swine Fever introduction from Eastern Asia is almost as high as from Eastern Europe | 2021 | Schweiz Arch Tierheilkd | Case study | Entry and exposure |
| Animal health and food safety risk assessments | 2021 | Rev Sci Tech | Review | N/A |
| A risk assessment of equine piroplasmosis entry, exposure and consequences in the UK | 2022 | Equine Vet J | Case study | Entry, Exposure & Consequence |

**Table S2: Articles meeting the selection criteria for further analysis by host species, pathogenic agent, country, route of spread and inputs/outputs**

| **Authors** | **Year** | **Host species** | **Agent** | **Country/Region** | **Route of spread** | **Inputs** | **Outputs** |
| --- | --- | --- | --- | --- | --- | --- | --- |
| McElvaine M.D., McDowell R.M., Fite R.W., Miller L. | 1993 | N/A | Foot and Mouth disease virus | USA | Garbage on cruise ships | Evidence, assumptions | Risk of entry of FMDV to domestic animals via garbage |
| Osborne C.G., McElvaine M.D., Ahl A.S., Glosser J.W. | 1995 | N/A | N/A | N/A | N/A | N/A | N/A |
| Ahl A.S. | 1996 | N/A | N/A | N/A | N/A | N/A | N/A |
| MacDiarmid S.C., Thompson E.J. | 1997 | N/A | Various | N/A | Import of sheep and goat meat | Evidence | Probability of introduction via contaminated meat |
| Leighton F.A. | 2002 | N/A | N/A | N/A | N/A | N/A | N/A |
| Gravenor M.B., Kao R.R. | 2003 | N/A | Bovine spongiform encephalopathy agent | N/A | N/A | N/A | N/A |
| Peeler EJ, Thrush MA. | 2004 | Atlantic salmon | *Gyrodactylus salaris* | United Kingdom | Mechanical, live fish and gametes, fish carcasses | Evidence, assumptions | Relative risk of various pathways of introduction |
| Yamamoto T., Kobayashi S., Nishiguchi A., Nonaka T., Tsutsui T. | 2006 | Cattle | Bovine spongiform encephalopathy agent | Japan | Contaminated fertiliser derived from slaughterhouse sludge | Evidence, assumptions | Identification of mitigation measures to prevent entry |
| Suedel B.C., Bridges T.S., Kim J., Payne B.S., Miller A.C. | 2007 | N/A | Aquatic nuisance species | United States | N/A | N/A | N/A |
| Peeler E.J., Murray A.G., Thebault A., Brun E., Giovaninni A., Thrush M.A. | 2007 | N/A | Aquatic pathogens | N/A | N/A | N/A | N/A |
| Suk J., Lyall C., Tait J. | 2008 | N/A | N/A | United Kingdom/Africa | N/A | Surveys | N/A |
| St-Hilaire, S., Thrush, M., Tatarian, T., Prasad, A. & Peeler, E. | 2009 | Amphibians | *Batrachochytrium denrobatidis* | N/A | Anthropogenic spread between water bodies | Responses to questions | Assist in reduction of risk of transfer of pathogen |
| Peeler EJ, Thrush MA | 2009 | Rainbow trout | Viral haemorrhagic septicaemia, infectious haematopoietic necrosis and *Gyrodactylus salaris* | United Kingdom | Spread via live fish transporters | Evidence, assumptions | Basis on which to consider policy development to reduce the risk of disease introduction via the movement of live fish transporters to an acceptable level |
| Peeler, E.J., Afonso, A., Berthe, F., Brun, E., Rodgers, C.J., Roque, A., Whittington, R. & Thrush, M.A. | 2009 | Fish | Epizootic Haematopoietic Necrosis Virus | England and Wales | Mechanical spread via importation of non-susceptible fish species | Evidence, assumptions | identification of risk mitigation measures |
| Jori F., Vosloo W., Du Plessis B., Bengis R., Brahmbhatt D., Gummow B., Thomson G.R. | 2009 | Livestock | Foot and Mouth disease virus | South Africa | Contact between infected wildlife and livestock | Evidence, assumptions | Framework for estimating the risk of disease outbreaks at the wildlife/livestock interface |
| Hayama Y., Kobayashi S., Nishida T., Nishiguchi A., Tsutsui T. | 2010 | Horses | Equine infectious disease | Japan | Horse movements | Survey results | Identification of risk groups for targeted surveillance and mitigation measures |
| Paton D.J., Sinclair M., Rodríguez R. | 2010 | Cattle | Foot and Mouth disease virus | N/A | Consumption of infected deboned meat products | Evidence, assumptions | Recommendations on mitigations |
| Heller J, Kelly L, Reid SW, Mellor DJ. | 2010 | Dogs | Meticillin-resistant *staphylococcus aureus* | N/A | Contact with infected humans, environment | Evidence, assumptions | Relative risk of infection in pet dogs between pathways |
| Gale P., Estrada-Peña A., Martinez M., Ulrich R.G., Wilson A., Capelli G., Phipps P., De La Torre A., Muñoz M.J., Dottori M., Mioulet V., Fooks A.R. | 2010 | Livestock | Crimean-Congo haemorrhagic fever virus | Europe | Contact with infected ticks, wildlife and livestock | Evidence, assumptions | Identification of risk pathways |
| Wieland B., Dhollander S., Salman M., Koenen F. | 2011 | Swine | African swine fever virus | Europe | Domestic pigs, wild boar, animal products, ticks, fomites, environmental contamination | Evidence, assumptions | Effect of mitigation measures on risk pathway |
| Abdo-Salem S, Waret-Szkuta A, Roger F, Olive MM, Saeed K, Chevalier V. | 2011 | Small ruminants | Rift Valley fever virus | Yemen | Importation of infected small ruminants | Evidence, assumptions | Assessment of likelihood of introduction and options to reduce the likelihood |
| Dufour B., Plée L., Moutou F., Boisseleau D., Chartier C., Durand B., Ganière J.P., Guillotin J., Lancelot R., Saegerman C., Thébault A., Hattenberger A.M., Toma B. | 2011 | N/A | N/A | N/A | N/A | N/A | N/A |
| De Vos C.J., Conraths F.J., Adkin A., Hallgren G.S., Paisley L.G. | 2011 | N/A | N/A | N/A | N/A | N/A | N/A |
| Salman M., Silano V., Heim D., Kreysa J. | 2012 | Cattle | Bovine Spongiform Encephalitis agent | Europe | Contaminated feedstuff or live cattle | Evidence, assumptions | Assessment of BSE status of countries |
| Corbellini L.G., Pellegrini D.C.P., Dias R.A., Reckziegel A., Todeschini B., Bencke G.A. | 2012 | Avian | Highly pathogenic avian influenza virus | Brazil | Contact with infected wild birds | Evidence, assumptions | Risk assessment was used to demonstrate the need for a prevention strategy plan |
| Peel A.J., Hartley M., Cunningham A.A. | 2012 | Amphibians | *Batrachochytrium dendrobatidis* | United Kingdom | Import of live infected amphibians | Evidence, assumptions | Identification of required risk management measures |
| Snary EL, Ramnial V, Breed AC, Stephenson B, Field HE, Fooks AR. | 2012 | N/A | Henipavirus | United Kingdom | Import of infected people, animals and contaminated food products | Evidence, assumptions | Estimate of the likelihood of entry of henipavirus via different pathways |
| EFSA Scientific Committee | 2012 | N/A | N/A | N/A | N/A | N/A | N/A |
| Sassnau R., Genchi C. | 2013 | Dogs | *Dirofilaria repens* | Germany | Movement of infected dogs | Evidence, assumptions | Risk of endemicity as a result of movement of infected dogs and climate change |
| Alemayehu G., Zewde G., Admassu B. | 2013 | Cattle | Lumpy skin disease virus | Ethiopia | Contact with infected animals/arthropods or contaminated equipment | Evidence, assumptions | Economic loss due to death/rejection of animals as a result of infection |
| Hill A., Brouwer A., Donaldson N., Lambton S., Buncic S., Griffiths I. | 2013 | Swine | Various | United Kingdom | Ability to detect disease at post-mortem | Evidence, assumptions | Risk/benefit assessment for visual only meat inspection |
| Hill A.A., Horigan V., Clarke K.A., Dewé T.C.M., Stärk K.D.C., O'Brien S., Buncic S. | 2014 | Cattle, sheep, goats, deer | Various | United Kingdom | Ability to detect disease at post-mortem | Evidence, assumptions | Risk assessment for visual only meat inspection |
| Gale P., Goddard A., Breed A.C., Irvine R.M., Kelly L., Snary E.L. | 2014 | Avian | Highly pathogenic avian influenza virus | Great Britain, the Netherlands and Italy | Migration of infected wild birds | Evidence, assumptions | Can be used to inform surveillance activities through focusing on certain species and migratory pathways. |
| Chazya R, Muma JB, Mwacalimba KK, Karimuribo E, Mkandawire E, Simuunza M. | 2014 | Goats | Peste des petits ruminants virus | Zambia | Movement of infected animals | Evidence, assumptions, questionnaires | Consequence of importing infected animals at village, district and national level |
| Pearce, F. M., Oidtmann, B. C., Thrush, M. A., Dixon, P. F., & Peeler, E. J. | 2014 | Fish | Viral haemorrhagic septicaemia virus | England and Wales | Processing of infected imported rainbow trout carcasses from Europe | Evidence, assumptions | Identification of steps in the risk pathway where defined mitigation measures could be implemented |
| Wieland B., Batsukh B., Enktuvshin S., Odontsetseg N., Schuppers M. | 2015 | Livestock | Foot and Mouth disease virus | Mongolia | Animal/people movements, contaminated environment, feed and fomites | Evidence, assumptions, expert opinions | Local understanding of the risk of disease resulting in ideas for prevention, control and eradication |
| Peeler E.J., Reese R.A., Thrush M.A. | 2015 | N/A | N/A | N/A | N/A | N/A | N/A |
| Crotta M., Ferrari N., Guitian J. | 2016 | Atlantic salmon | Anisakid larvae | N/A | Infected hosts or contaminated feed | Evidence, assumptions | Risk of commercialization of salmon infested by viable larvae |
| Kyyrö J., Sahlström L., Lyytikäinen T. | 2017 | Swine | African swine fever virus | Finland | Domestic pigs, wild boar, animal products, ticks, fomites, environmental contamination | Evidence, assumptions | High overall risk of ASF entry and consequence into Finland |
| Dalziel A.E., Sainsbury A.W., McInnes K., Jakob-Hoff R., Ewen J.G. | 2017 | N/A | N/A | N/A | N/A | N/A | N/A |
| Kelly L., Kosmider R., Gale P., Snary E.L. | 2018 | N/A | N/A | N/A | N/A | N/A | N/A |
| Auty H., Mellor D., Gunn G., Boden L.A. | 2019 | Livestock | Foot and Mouth disease virus | United Kingdom | Countryside activities | Evidence, assumptions | Relative likelihood of different recreational activities causing new infected premises |
| Amanatin A., Sudarnika E., Lukman D.W., Wibawan I.W.T. | 2019 | Dogs | Rabies virus | Indonesia | Movement of hunting dogs | Evidence, questionnaire, observation | Identification where risk management can be focussed |
| Cripps J.K., Pacioni C., Scroggie M.P., Woolnough A.P., Ramsey D.S.L. | 2019 | Deer | Various | Australia | Transmission of disease from wild cervids to livestock | Evidence | Identification of pathogens posing a risk to the livestock industry |
| Horigan V, Gale P, Adkin A, Brown I, Clark J, Kelly L. | 2019 | Avian | Highly pathogenic avian influenza virus | United Kingdom | Contact with contaminated equipment | Evidence, assumptions | Comparison of risk of infection between dismantling equipment or not |
| Cabral M., Taylor R., de Vos C.J. | 2019 | N/A | N/A | N/A | N/A | N/A | N/A |
| Rinchen S., Tenzin T., Hall D., Cork S. | 2020 | Dogs | Rabies virus | Bhutan | Movement of stray/pet dogs and cattle | Evidence, assumptions, expert opinion | Highlights the importance of the current risk mitigation measures |
| Squarzoni-Diaw C., Arsevska E., Kalthoum S., Hammami P., Cherni J., Daoudi A., Karim Laoufi M., Lezaar Y., Rachid K., Seck I., ould elmamy B., Yahya B., Dufour B., Hendrikx P., Cardinale E., Muñoz F., Lancelot R., Coste C. | 2020 | Livestock | Foot and Mouth disease virus | Tunisia | Live animal movements | Evidence, assumptions, expert opinion | Illustration of how risk assessment can be used as a decision support tool for risk-based surveillance |
| de Vos CJ, Taylor RA, Simons RRL, Roberts H, Hultén C, de Koeijer AA, Lyytikäinen T, Napp S, Boklund A, Petie R, Sörén K, Swanenburg M, Comin A, Seppä-Lassila L, Cabral M, Snary EL. | 2020 | Swine | African swine fever virus | the Netherlands, Finland, Germany | Domestic pigs, wild boar, animal products, ticks, fomites, environmental contamination | Evidence, assumptions | Cross validation of risk assessment tools |
| Islam SS, Akwar H, Hossain MM, Sufian MA, Hasan MZ, Chakma S, Meeyam T, Chaisowwong W, Punyapornwithaya V, Debnath NC, Brum E, Pichpol D. | 2020 | Avian | Highly pathogenic avian influenza virus | Bangladesh | Live/dead birds, fomites, animal products, animal by-products | Evidence, assumptions, expert opinions and observations | Can be used to formulate effective risk reduction measures |
| Horigan V, Gale P, Adkin A, Konold T, Cassar C, Spiropoulos J, Kelly L. | 2020 | N/A | Prion disease agent | United Kingdom | Live animals/animal products | Evidence, assumptions | Aggregated probability of entry |
| Common S.M., Shadbolt T., Walsh K., Sainsbury A.W. | 2021 | Bats | SARS-CoV-2 | England | Contact with infected people through field activities | Evidence, assumptions | Suggested disease risk management measures |
| Babayani ND, Thololwane OI. | 2021 | Cattle | Foot and Mouth disease virus (FMDV) | Botswana | Wildlife | Evidence, assumptions, expert opinion | Suggested disease risk management measures |
| Friker B, Schüpbach G. | 2021 | Swine | African swine fever virus | Switzerland | wildlife, live animal/animal products, ticks, fomites, humans | Evidence, assumptions, expert opinion | Comparison of probability of introduction of disease between Eastern Europe and Eastern Asia |
| Makita K. | 2021 | N/A | N/A | N/A | N/A | N/A | N/A |
| Coultous RM, Sutton DGM, Boden LA. | 2022 | Horses | *Theileria equi* and/or *Babesia caballi* | United Kingdom | Live animals/blood products/ ticks | Evidence, assumptions | Suggested disease risk management measures |
